# Molecular Dynamics of Lysozyme Amyloid Polymorphs Studied by Incoherent Neutron Scattering

**DOI:** 10.3389/fmolb.2021.812096

**Published:** 2022-01-17

**Authors:** Tatsuhito Matsuo, Alessio De Francesco, Judith Peters

**Affiliations:** ^1^ Univ. Grenoble Alpes, CNRS, LiPhy, Grenoble, France; ^2^ Institut Laue-Langevin, Grenoble, France; ^3^ Institute for Quantum Life Science, National Institutes for Quantum Science and Technology, Tokai, Japan; ^4^ CNR-IOM and INSIDE@ILL C/O Operative Group in Grenoble (OGG), Grenoble, France; ^5^ Institut Universitaire de France, Paris, France

**Keywords:** elastic incoherent neutron scattering, quasi-elastic neutron scattering, lysozyme amyloidosis, polymorphism, amyloid fibrils, protein dynamics

## Abstract

Lysozyme amyloidosis is a hereditary disease, which is characterized by the deposition of lysozyme amyloid fibrils in various internal organs. It is known that lysozyme fibrils show polymorphism and that polymorphs formed at near-neutral pH have the ability to promote more monomer binding than those formed at acidic pH, indicating that only specific polymorphs become dominant species in a given environment. This is likely due to the polymorph-specific configurational diffusion. Understanding the possible differences in dynamical behavior between the polymorphs is thus crucial to deepen our knowledge of amyloid polymorphism and eventually elucidate the molecular mechanism of lysozyme amyloidosis. In this study, molecular dynamics at sub-nanosecond timescale of two kinds of polymorphic fibrils of hen egg white lysozyme, which has long been used as a model of human lysozyme, formed at pH 2.7 (LP27) and pH 6.0 (LP60) was investigated using elastic incoherent neutron scattering (EINS) and quasi-elastic neutron scattering (QENS). Analysis of the EINS data showed that whereas the mean square displacement of atomic motions is similar for both LP27 and LP60, LP60 contains a larger fraction of atoms moving with larger amplitudes than LP27, indicating that the dynamical difference between the two polymorphs lies not in the averaged amplitude, but in the distribution of the amplitudes. Furthermore, analysis of the QENS data showed that the jump diffusion coefficient of atoms is larger for LP60, suggesting that the atoms of LP60 undergo faster diffusive motions than those of LP27. This study thus characterizes the dynamics of the two lysozyme polymorphs and reveals that the molecular dynamics of LP60 is enhanced compared with that of LP27. The higher molecular flexibility of the polymorph would permit to adjust its conformation more quickly than its counterpart, facilitating monomer binding.

## Introduction

Lysozyme amyloidosis is an autosomal dominant hereditary systemic amyloidosis, which was first identified in 1993 (Pepys et al., 1993). Although it is a rare disease, it causes serious symptoms such as disruption of the kidney function, gastrointestinal and/or hepatic spontaneous hemorrhage, which are often fatal, and is characterized by deposition of lysozyme amyloid fibrils in various internal organs. The main cause of this disease is the mutation in lysozyme and so far, there have been about 10 mutations detected (Röcken et al., 2006; Iqbal et al., 2019). However, despite a huge accumulation of knowledge of the lysozyme molecules including disease-causing mutants (Dumoulin et al., 2006), the molecular mechanism of the pathogenesis of lysozyme amyloidosis is not yet fully understood.

Detailed clinical observations on the three mutants of lysozyme (I56T, D67H, and L84S) have shown that each mutation shows different phenotypes or symptoms in the patients (Gillmore et al., 1999; Nasr et al., 2017), suggesting that the fibrils formed from these mutants of lysozyme show distinct physico-chemical properties and thus have different detrimental effects on cells. This puts an emphasis on the importance of the investigation of the relationship between properties of lysozyme fibrils and degrees of cytotoxic effects. It is, however, often difficult to obtain sufficient amounts of disease-related mutants of human lysozyme for biophysical measurements. Therefore, polymorphic nature of fibrils produced from the wild-type lysozyme has widely been exploited. In fact, lysozyme forms amyloid fibrils with different morphology depending on fibrillation conditions such as pH, temperature, salt concentration, etc. Furthermore, due to its highly homologous nature, hen egg white lysozyme (HEWL), a 14.3 kDa bacteriolytic enzyme with 129 residues, has often been used instead of human lysozyme, as a model system to study the molecular mechanism of amyloid fibril formation and eventually the lysozyme amyloidosis. It is now accepted that lysozyme amyloid fibrils formed at near-neutral pH are characterized by aggregates of shorter fibrils and stronger cytotoxic effects while those formed at acidic pH are characterized by longer fibrils and weaker cytotoxic effects (Mossuto et al., 2010; Mocanu et al., 2014; Sulatskaya et al., 2017; Rahimi Araghi and Dee, 2020). Polymorphic fibrils have been identified in the tissues of patients in several amyloidosis such as Alzheimer’s disease or AL amyloidosis (Lu et al., 2013; Annamalai et al., 2016; Fitzpatrick et al., 2017). Although lysozyme polymorphs have not yet been detected in patients, lysozyme polymorphism is also likely to occur in the patients because lysozyme fibrils are found in various internal organs, i.e., in different chemical environments. Therefore, as a first step to ultimately understand the molecular mechanism of cytotoxicity and of amyloidosis including lysozyme amyloidosis, it is important to investigate the properties of lysozyme polymorphs in detail. In particular, lysozyme polymorphs formed at acidic pH and at near-neutral pH have been paid much attention since it was originally shown that these polymorphs induce different levels of cytotoxicity (Mossuto et al., 2010).

Recently, intriguing features of the lysozyme polymorphs have been revealed by cross-seeding measurements (Rahimi Araghi and Dee, 2020). It has been shown that when lysozyme polymorphs are formed at near-neutral pH from both monomers and short fragments of fibrils (called “seeds”) preformed at acidic pH, the majority of the resultant fibrils shows a similar morphology as those formed at near-neutral pH. On the other hand, when fibrils are formed at acidic pH from both monomers and seeds preformed at near-neutral pH, the resultant fibrils again show the characteristics specific to those formed at near-neutral pH. These findings have led the authors to conclude that the polymorph formed at near-neutral pH has the ability to recruit additional monomers more easily and independently of solution conditions. Such finding is probably due to faster configurational diffusion, i.e., enhanced molecular dynamics, in order to access the polymorph-specific particular conformation (Rahimi Araghi and Dee, 2020). It further points to the importance of the intrinsic dynamical behavior of the polymorphs in determining whether they become the dominant species in a given chemical environment. Understanding the dynamical features of polymorphs is thus crucial not only for deepening our understanding of amyloid polymorphism, but also for ultimately understanding the mechanism of cytotoxicity and pathogenesis of lysozyme amyloidosis. However, it still remains unclear whether there exist differences in molecular dynamics of lysozyme polymorphs formed at acidic or near-neutral pH and what kind of differences there are between the internal motions of the polymorphs if any. In particular, since side chains of the polymorphs interact directly with those of other polymorphs or monomers, it is important to focus on the side chain mobility of lysozyme polymorphs.

Incoherent neutron scattering (iNS) is a powerful technique to investigate molecular dynamics of biomacromolecules at the pico- to nano-second timescale and at the Ångström length scale. Since the incoherent scattering cross-section of the hydrogen atom is ∼40 times larger than that of any other atoms found in biomacromolecules and its isotope deuterium, iNS permits to observe the motions of hydrogen atoms contained in biomacromolecules. In the case of proteins, the motions observed by iNS reflect those of chemical groups such as amino acid side chains or backbones, to which hydrogen atoms are bound. Moreover, because the hydrogen atoms are distributed quasi-uniformly throughout a protein molecule, iNS provides the dynamical information on the motions of hydrogen atoms averaged over the whole molecule. In this study, two iNS techniques were employed: elastic incoherent neutron scattering (EINS) and quasi-elastic neutron scattering (QENS) (Gabel et al., 2002). EINS provides information on the amplitudes of atomic motions (Zaccai, 2000), while QENS yields information on diffusive motions such as frequency and geometry of atomic motions (Bée, 1988). In amyloid research, EINS and QENS have been employed to investigate the dynamical change of amyloidogenic proteins upon fibril formation such as concanavalin A (Schirò et al., 2012), lysozyme (Fujiwara et al., 2013) and α-synuclein (Bousset et al., 2014; Fujiwara et al., 2016), or the dynamical change of hydration water around tau proteins upon fibril formation (Fichou et al., 2015). These studies have succeeded to identify the dynamical behavior specific to monomers or fibrils, thereby advancing our understanding of the molecular mechanism of amyloid fibril formation in terms of molecular dynamics. On the other hand, the molecular dynamics of fibrils with different morphology formed from identical protein monomers has not yet been studied despite its importance with regards to pathogenesis of amyloidosis.

In this study, we investigated the sub-ns dynamics of two kinds of D_2_O-hydrated samples of HEWL polymorphic fibrils, which were formed at pH 2.7 and pH 6.0, using EINS and QENS. In order to analyze the local atomic motions in detail by suppressing large scale motions, hydrated powder samples were employed. It was found that the polymorphs formed at pH 6.0 contain a major fraction of atoms undergoing diffusive motions with larger amplitudes, and undergo faster local diffusive motions than those at pH 2.7. These observations are discussed in relation with the physicochemical nature of the polymorphs.

## Materials and Methods

### Sample Preparation

Lysozyme fibrils were formed as previously reported (Mocanu et al., 2014) with longer incubation time to ensure aggregate formation: HEWL lyophilized powder (L6876-1G, Sigma-Aldrich) was incubated in a buffer containing 70 mM glycine (pH 2.7) and 80 mM NaCl, or containing 20 mM Na_2_HPO_4_ (pH 6.0) and 80 mM NaCl for 2.5 h at 65°C with constant stirring at the speed of 1,200 rpm using a thermomixer HCM100-Pro (DLAB Scientific). Hereafter, the lysozyme polymorphs formed at pH 6.0 and pH 2.7 are denoted as LP60 and LP27, respectively. The fibril solutions were subsequently dialyzed against D_2_O to remove buffer components, followed by lyophilization. The lyophilized samples were rehydrated for ∼72 h until the hydration ratio reached 0.4 g D_2_O/g protein in a desiccator with a Petri dish filled with D_2_O at the bottom of it. The hydration level was monitored by weighing the samples. Both samples were prepared in parallel in order to obtain directly comparable samples. For neutron scattering measurements, the rehydrated powder samples were put into flat aluminum cells (3 × 4 cm^2^) of 1 mm thickness and sealed with indium wire. The weight of the cells was checked before and after the neutron scattering measurements to control that the hydration level was maintained, and there was no loss of weight during the measurements.

### Attenuated Total Reflectance Fourier Transform Infrared (ATR-FTIR) Spectroscopy

Characterization of the polymorphs was first conducted by ATR-FTIR. ATR-FTIR spectra were recorded in the amide I region (1,580–1,710 cm^−1^) at a resolution of 2 cm^−1^ using the FT/IR-4600 spectrometer with a single-reflection ATR accessory with a diamond crystal (JASCO, Tokyo, Japan). 10 μl of samples in D_2_O was put on the pedestal and 64–128 interferograms were averaged. In addition to the two kinds of polymorphs, HEWL monomers lyophilized powder (L6876-1G, Sigma-Aldrich) dissolved in D_2_O were also measured as a control. The spectra of D_2_O were subtracted from those of the samples. The deconvolution of the measured FTIR spectra into Gaussian functions was carried out using IGOR Pro software (WaveMetrics, Lake Oswego, OR, United States) so that the discrepancy in both the FTIR spectra and their second derivatives between measured and fitted curves was minimized.

### Atomic Force Microscopy (AFM)

Further, AFM images of the lysozyme polymorphs were taken using a Cypher S AFM instrument (Asylum Research, Santa Barbara, CA, United States). 10 μl of samples in D_2_O was placed on a mica plate and then left for 5 min to adsorb proteins on its surface, followed by rinsing with ultrapure water and 2–3 h of air-drying before recording images.

### Elastic Incoherent Neutron Scattering (EINS)

EINS measurements were carried out at the Collaborative Research Group (CRG) thermal neutron back-scattering spectrometer IN13 at the Institut Laue-Langevin (Grenoble, France) (Natali et al., 2008). The sample weights in the aluminum cells were 0.1671 and 0.1586 g for LP60 and LP27, respectively. EINS spectra were measured on these hydrated powder samples in the temperature range of 20–310 K at a wavelength 2.23 Å (16 meV) with the energy resolution of 8 μeV, corresponding to the time window of ∼160 ps. The instrumental configuration covers a broad range of momentum transfers in the range of 0.2 Å^-1^ < Q < 4.9 Å^-1^, which correspond to amplitudes of 1.28 Å < d < 31.4 Å (Q = 2π/d). The heating rates were set to be 0.3 K/min, 0.15 K/min, and 0.077 K/min for 20–100 K, 100–200 K, and 200–310 K, respectively, because the elastic intensity decreases as the temperature increases due to the excitation of diffusive atomic motions. The data reduction was carried out using the LAMP package (Richard et al., 1996). The spectra of the empty cell were subtracted from those of samples with an appropriate coefficient calculated from the transmission measurement, which showed that the transmission of all the samples was >94%, and thus contribution of multiple scattering to the measured spectra was negligible. The spectra of the vanadium slab with 2 mm thickness were used for the correction of the detector efficiency. Fitting of the spectra was done using IGOR Pro software (WaveMetrics, Lake Oswego, OR, United States).

In the analysis of the EINS data, the mean square displacement (MSD) of atomic motions was first extracted from the measured EINS curves using the following Gaussian approximation (Rahman et al., 1962):

$$S\left(Q,\;\pm \Delta E\right)=S\left(0\right)\exp\left(-\frac{1}{3}Q^{2}\langle u^{2}\rangle \right),$$ (1)

where ΔE denotes the energy resolution, S(0) is the incident scattering intensity at Q = 0 [Å^−1^] and $\langle u^{2}\rangle$ is the MSD of atomic motions. Note that the MSD values reported in this study are those relative to the MSD value at the lowest temperature (24 K) because the EINS curves were normalized by that at 24 K. While the fitting using Eq. 1 is rigorously valid under the condition of $Q^{2}\langle u^{2}\rangle \mathrm{<1}\mathrm{.3}$ , it is known that for protein dynamics the Gaussian approximation is valid for $Q^{2}\langle u^{2}\rangle <4$ (Stadler et al., 2013). Furthermore, it is recommended to include data points at higher Q values as long as the data points follow the Gaussian behavior in order to obtain more reliable and consistent results (Zeller et al., 2018). The MSD values in this study were thus evaluated in the range of $Q^{2}\langle u^{2}\rangle <\mathrm{2.4}$ .

In addition to the Gaussian approximation, a mean-square atomic position fluctuation (MSPF) analysis (Peters and Kneller, 2013) was also carried out. Unlike the MSD analysis, the MSPF analysis uses all the available data points in the EINS curves, so that more detailed dynamical information can be extracted. Here, a motional heterogeneity of the amplitudes of the atomic motions is introduced in the form of a Gamma distribution. It is known that a “shifted” Gamma distribution reproduces quite well the distributions of atomic position fluctuations, which are calculated from the trajectory of both the molecular dynamics simulation and normal-mode analysis (Kneller and Hinsen, 2009), suggesting that a “shifted” Gamma distribution is a reliable measure of the distribution of amplitudes of atomic motions in protein molecules. In practice, since the value of the “shift” cannot be determined from the experimental data, a normal Gamma distribution has been employed (Peters and Kneller, 2013; Zeller et al., 2018). The elastic scattering intensity in the MSPF analysis is written as (Peters and Kneller, 2013),

$$S\left(Q,\;\pm \Delta E\right)=S\left(0\right){\left(1+\frac{Q^{2}\langle r^{2}\rangle }{\beta }\right)}^{-\beta }\text{,}$$ (2)

where $\langle r^{2}\rangle \;$ is the MSPF of the atomic motions and β is the measure of the motional heterogeneity. The distribution of the atomic position fluctuations is described by the Gamma distribution,

$$p\left(\lambda ,\beta \right)=\frac{\beta \exp\left(-\beta \lambda \right){\left(\beta \lambda \right)}^{\beta -1}}{\Gamma \left(\beta \right)}\;\;\;\;\left(0<\beta <\;\infty \right)\text{,}$$ (3)

where $\lambda =r^{2}/\langle r^{2}\rangle$ is a dimensionless value and denotes the squared atomic position fluctuation relative to the MSPF. In the limit of β→ ∞ , the Gaussian approximation is retrieved. Thus, for a constant MSPF value, the increase in  β means the decrease in the motional heterogeneity.

### Quasi-Elastic Neutron Scattering (QENS)

**FIGURE 1:**
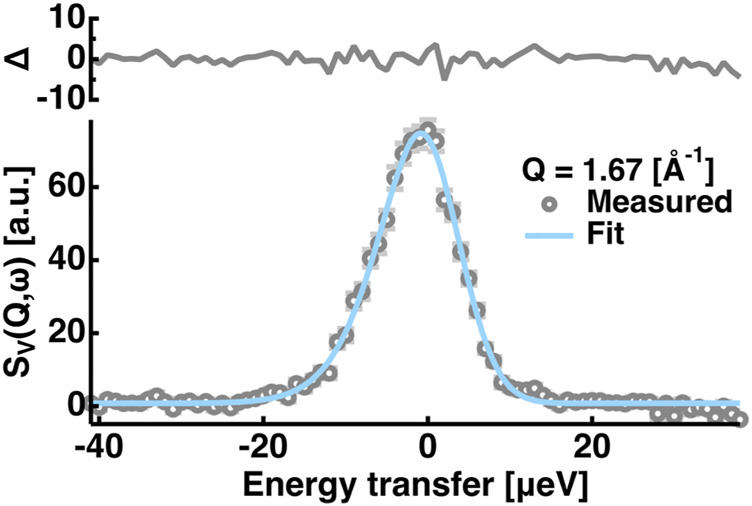
Example of the fitting of the QENS spectra of the vanadium. Those at Q = 1.67 [Å^−1^] are shown. Grey open circles denote the measured data points and the cyan solid line denotes the fit. The residuals (Δ) of the fitting are shown in the upper side of the panel. Error bars are shown in light grey and they are within the symbols if not shown.

In addition to the EINS measurements, QENS measurements were also carried out using IN13. New samples were prepared in the same way as above and the sample weights in aluminum cells were 0.1624 and 0.1644 g for LP60 and LP27, respectively. The wavelength of the incident neutron beam was varied by cooling or heating the monochromator of the instrument. The QENS spectra were measured in the energy transfer range of −80 μeV < ΔE < +85 μeV at 300 K. The procedures for the data reduction of the measured QENS data were the same as those described above. The QENS spectra of the vanadium were used as resolution functions, which show a slight asymmetry in terms of the energy transfer. To take this asymmetry into account, the measured scattering intensity of vanadium S_V_(Q, ω) as a function of momentum transfer Q and energy transfer ℏω was fitted by the following equation (Armstrong et al., 2011):

$$S_{V}\left(Q,\;\omega \right)=A\left[H\left(\hbar \omega_{0}-\hbar \omega \right)\exp\left(-\frac{{\left(\hbar \omega -\hbar \omega_{0}\right)}^{2}}{2\sigma^{2}+\xi \left|\hbar \omega -\hbar \omega_{0}\right|}\right)+H\left(\hbar \omega -\hbar \omega_{0}\right)\exp\left(-\frac{{\left(\hbar \omega -\hbar \omega_{0}\right)}^{2}}{2\sigma^{2}}\right)\right]+B\text{,}$$ (4)

where A is the amplitude, $\hbar \omega_{0}\;$ is the elastic peak position, σ is the Gaussian standard deviation, ξ is the asymmetry parameter, B is the background, and H is a Heaviside function. A Gaussian function is retrieved when ξ=0 . An example of the fitting of the vanadium spectra is shown in Figure 1. It was found that ξ **=** 2.77, which is in line with the previously reported value of 3.29 (Armstrong et al., 2011).

The obtained QENS spectra arising from proteins are fitted by the following equation:

$$S\left(Q,\;\omega \right)=C\left(Q\right)\left[A_{0}\left(Q\right)\delta \left(\omega \right)+\left(1-A_{0}\left(Q\right)\right)L\left(Q,\;\omega \right)\right]⊗R\left(Q,\;\omega \right)+B\left(Q\right)\text{,}$$ (5)

Where C(Q)  is the scaling factor including the Debye-Waller factor, $\;A_{0}\left(Q\right)$ is the elastic incoherent structure factor (EISF), R(Q, ω) is the resolution function, and B(Q) is the background. L(Q, ω)  is the Lorentzian function, which is written as,

$$L\left(Q,\;\omega \right)=\;\frac{1}{\pi }\frac{\Gamma \left(Q\right)}{\omega^{2}+\Gamma {\left(Q\right)}^{2}},$$ (6)

where Γ(Q) is the width of the Lorentzian function. In general, the fitting of the QENS spectra is carried out separately at each Q value. However, judging from the S/N ratio of the present QENS data, a global fitting approach, where the spectra at all the Q values are fitted at once using Eqs 5–9 (see below), was employed in this study. This approach has the advantage of reducing the number of parameters in the fitting by assuming some physical models in advance while it increases the experimental data points available compared with the conventional fitting, leading to the determination of more reliable parameter values. The global fitting approach has previously been done in several studies and allowed detailed discussion on the system investigated (Schirò et al., 2015; Piazza et al., 2018; Al-Ayoubi et al., 2019). In the current global fitting, Γ(Q) was represented by a jump diffusion (Bée, 1988), which is described as

$$\Gamma \left(Q\right)=\;\frac{DQ^{2}}{1+DQ^{2}\tau }\text{,}$$ (7)

where τ and D denote the residence time and the jump diffusion coefficient, respectively. The EISF was described by a diffusion-inside-a-sphere model written as (Volino and Dianoux, 1980),

$$A_{0}\left(Q\right)=\left(1-\;p_{0}\right){\left(\frac{3j_{1}\left(Qa\right)}{Qa}\right)}^{2}+\;p_{0},$$ (8)

where $\;p_{0}$ is the immobile fraction of atoms, the motion of which is too slow to be observed by the energy resolution employed, $j_{1}\left(Qa\right)$ is the first-order spherical Bessel function of the first-kind, and a is the radius within which an atom can move. The background was represented as follows taking account of its possible Q-dependence,

B(Q)=sQ+t, (9)

**FIGURE 2:**
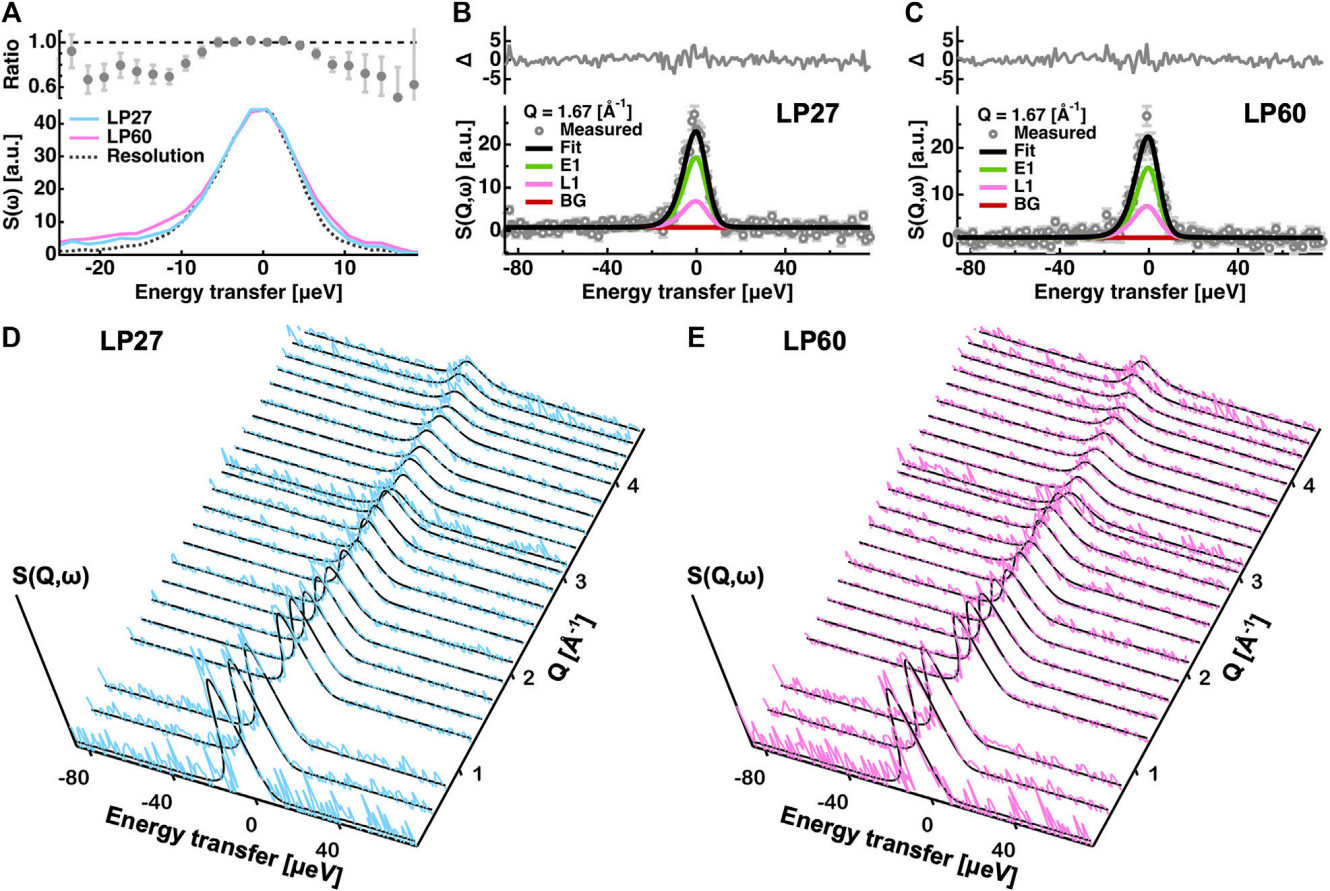
Analysis of the QENS spectra. **(A)**: Comparison of the QENS spectra integrated over all the Q-range measured. Those of LP27 and of LP60 are denoted by cyan and magenta lines, respectively. The resolution function is represented by a dotted line. In the upper panel, the intensity ratio of LP27 to LP60 is shown. **(B,C)**: Examples of the fitting of the QENS spectra at Q = 1.67 [Å^−1^] of LP27 **(B)** and LP60 **(C)**. Experimental data points are denoted by grey open circles. The total fit (Fit), the elastic component (E1), the Lorentzian function (L1), and the background (BG) are denoted by black, green, magenta, and brown lines, respectively. In the upper panel, the residuals (Δ) between the measured and the fitting values are shown. Error bars are shown in light grey. **(D,E)**: Comparison of the obtained QENS spectra and the corresponding fits at all the Q values measured for LP27 **(D)** and LP60 **(E)**. The experimental QENS spectra in **(D,E)** are shown in cyan and magenta, respectively. The fits are shown by black lines.

Where  s and  t are parameters in the global fitting. It was found that the global fitting by the above equations resulted in the χ^2^ values of 1.2 and 1.1 for LP27 and LP60, respectively, with χ^2^ defined as

$$\chi^{2}=\frac{1}{N-1}\sum_{j=1}^{N}\sum_{i=1}^{M}{\left(\frac{S_{\exp}\left(Q_{j},\;\omega_{i}\right)-S_{sim}\left(Q_{j},\;\omega_{i}\right)}{\sigma_{i}}\right)}^{2}\text{,}$$ (10)

where N and M denote the number of Q values and the number of data points in the QENS spectra at one Q value, respectively, and $\sigma_{i}$ is the experimental error at the *i*th data points in the QENS spectra. Fitting examples at Q = 1.67 [Å^−1^] using Eq. 5 are shown in Figures 2B,C, and the comparisons of the measured spectra with the fits at all the Q-values are shown in Figures 2D,E for both the investigated samples. As expected from the χ^2^ values close to 1.0, the simulated curves fit the measured data points quite well. Another trial of the global fitting was also carried out by replacing Eq. 7 with

$$\Gamma \left(Q\right)=\;\frac{1}{\tau },$$ (11)

which describes conformational changes with a correlation time of  τ . Such Q^2^-independent behaviour was observed for fully or partially folded myoglobin (Stadler et al., 2015) and macromolecular motions in bacteria (Jasnin et al., 2008). However, this global fitting yielded χ^2^ values of 2.1 and 1.7 for LP27 and LP60, respectively, which are much worse than those obtained from the original fitting using Eq. 7. Furthermore, alternative descriptions of the EISF, e.g., a Gaussian distribution (Perez et al., 1999) or a lognormal distribution (Gibrat et al., 2008), did not affect the discussion below (data not shown). Therefore, the results obtained by the original fitting (Eqs 5–9) were chosen here to discuss the diffusive motions of the lysozyme polymorphs.

## Results

### Sample Characterization

**FIGURE 3:**
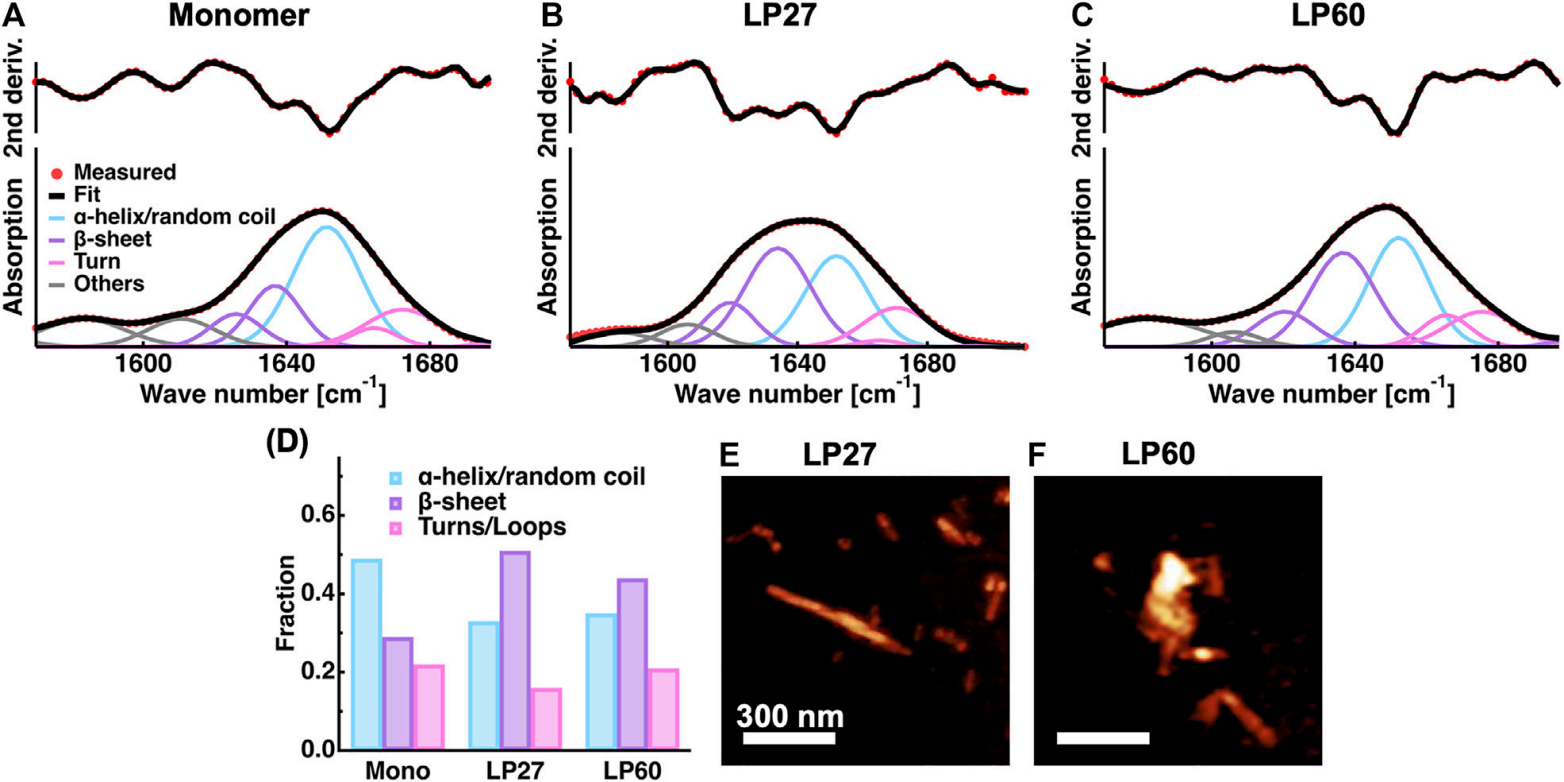
Sample characterization. **(A–C)**: ATR-FTIR spectra of HEWL monomers **(A)** and HEWL polymorphs formed at pH 2.7 [LP27; **(B)**] and at pH 6.0 [LP60; **(C)**]. The second derivatives of the spectra and the fitting results are also shown. Red filled circles denote the experimental data points and black solid lines denote the fits. The solid lines in cyan, purple, magenta, and grey represent the contributions from α-helices/random coils, β-sheets, turn structures, and other contributions, respectively. **(D)**: The secondary structure content of the samples estimated from the spectra shown in **(A–C)**. **(E–F)**: AFM images of LP27 **(E)** and LP60 **(F)** shown using the software Gwyddion (Nečas and Klapetek, 2012). The scale bars correspond to 300 nm.

In Figure 3, results from ATR-FTIR and AFM measurements are reported. It was found that the ATR-FTIR spectra and their second derivatives of LP27 and LP60 are distinct from each other, and at the same time distinct from those of monomers [Figures 3 (A–C)], suggesting that HEWL monomers are transformed into two different structures (polymorphs) depending on the fibrillation conditions. Figure 3D shows the secondary structure content of the monomers, LP27 and LP60. It is found that HEWL monomers consist of 49% α-helix/random coil, 29% β-sheet, and 22% turn structures, which is consistent with the literature values (49.5% α-helix/random coil, 28.6% β-sheet, and 21.8% turn structures) obtained from ATR-FTIR of HEWL monomers (Chaari et al., 2015). Regarding the LP27 and LP60, the α-helical content decreases and the β-sheet content drastically increases upon transition from monomers to LP27 and LP60. Furthermore, the fraction of the β-sheet is lower for LP60 than for LP27, which is a general trend in lysozyme amyloid fibrils formed at acidic and near-neutral pH (Mossuto et al., 2010; Mocanu et al., 2014). The AFM images of LP27 (Figure 3E) and LP60 (Figure 3F) show that LP27 is an isolated fibril while LP60 tends to be an aggregate of shorter fibrils. These morphological features are also consistent with previous findings (Mossuto et al., 2010; Mocanu et al., 2014; Rahimi Araghi and Dee, 2020).

### EINS Measurements

**FIGURE 4:**
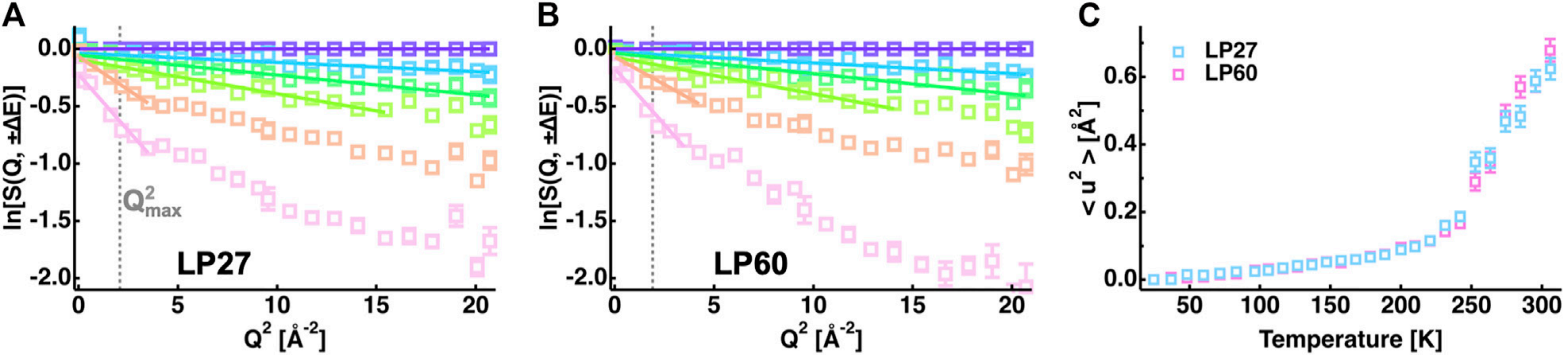
MSD analysis of the EINS curves. **(A,B)**: Q^2^-dependence of the logarithm of the EINS curves of LP27 **(A)** and LP60 **(B)** normalized by that at the lowest temperature of 24 K. The temperatures of these curves are 24, 96, 147, 200, 252, and 295 K (from top to bottom). The corresponding Gaussian fits are denoted by solid lines. For reference, in **(A,B)**, $Q_{\max}^{2}$ values, which are the square of the maximum Q values where the Gaussian approximation is strictly valid ( $Q_{\max}^{2}\langle u^{2}\rangle =1.3$ ), are denoted by grey dotted lines. $Q_{\max}^{2}$ was evaluated at the highest temperature of each sample, where the decay of the EINS curves with increasing Q is the steepest. **(C)**: MSD values as a function of temperature. Those of LP27 and of LP60 are denoted by open squares in cyan and magenta, respectively. In all the panels, error bars are within the symbols if not shown.

In order to characterize the atomic motions of LP27 and LP60, EINS measurements were carried out. For powder samples of lysozyme hydrated at 0.4 g D_2_O/g protein, the incoherent scattering cross section of lysozyme is calculated to be 3.23 × 10^−24^ cm^2^ per 1 g of lysozyme while that of the hydration water is 0.05 × 10^−24^ cm^2^ per 0.4 g D_2_O. Thus, the scattering contribution of lysozyme to the measured spectra is more than 98%, meaning that the scattering contribution of the hydration water is negligible in this study. First, the MSDs were evaluated from the EINS curves by the Gaussian approximation (see Eq. 1). Fitting examples are shown in Figures 4A,B, and the extracted MSD values are shown in Figure 4C as a function of temperature. It was found that the MSD values increase linearly at lower temperatures up to ∼130 K, then the slope rises up to ∼240 K, which is attributed to the excitation of the methyl group rotations (Wood et al., 2010). At higher temperatures above 240 K, the MSD values significantly increase with the temperature [so-called “dynamical transition” (Doster et al., 1989)], suggesting that diffusive local motions are activated. Although these features are consistent with those generally observed for hydrated protein powder samples (Gabel et al., 2002), the comparison of the MSD between LP27 and LP60 shows no significant differences between them, suggesting that the amplitudes of atomic motions averaged over all the hydrogen atoms in the protein are similar for both polymorphs.

**FIGURE 5:**
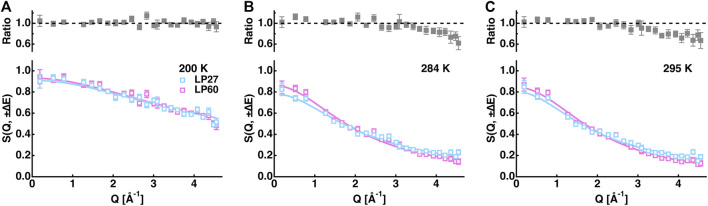
Comparison of the EINS curves between LP27 and LP60 at 200 K **(A)**, 284 K **(B)**, and 295 K **(C)**. The upper panels show the intensity ratio of LP60 to LP27. The cyan and magenta open squares denote the experimental data points of LP27 and LP60, respectively. The solid lines are the corresponding fits obtained by Eq. 2 in the main text.

**FIGURE 6:**
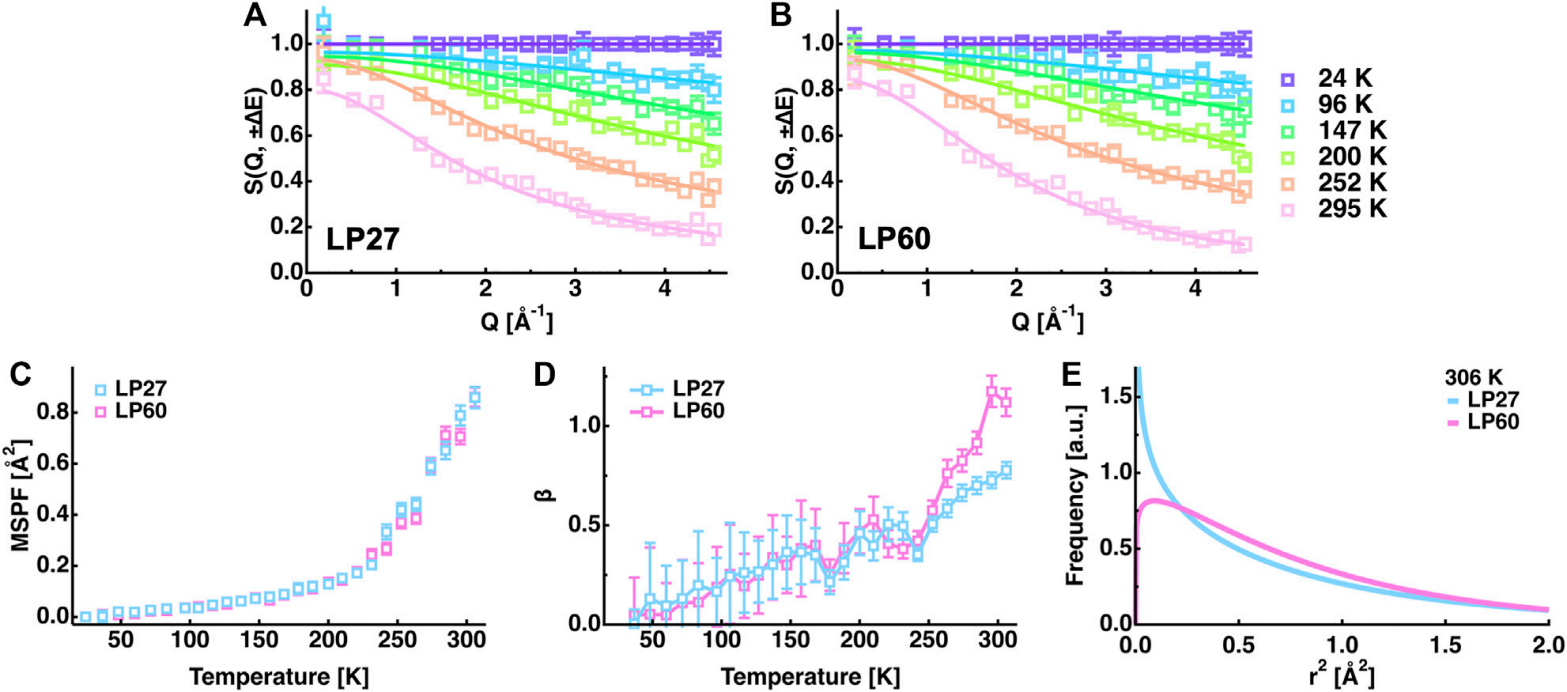
Summary of the MSPF analysis. **(A,B)**: EINS curves of LP27 **(A)** and LP60 **(B)** as a function of Q. The temperatures of these curves are 24, 96, 147, 200, 252, and 295 K (from top to bottom). The corresponding fits by Eq. 2 in the text are shown by solid lines. **(C)**: MSPF values as a function of temperature. Those of LP27 and of LP60 are denoted by cyan and magenta open squares, respectively. The same color notation is used in **(D,E)**. **(D)**: The measure of motional homogeneity (β) as a function of temperature. **(E)**: Distribution of the squared amplitudes of atomic motions at 306 K obtained by the MSPF analysis. In all the panels, error bars are within the symbols if not shown.

However, the EINS curves are not the same between LP27 and LP60 when compared beyond the Q range, which was used for the Gaussian approximation, as shown in Figure 5. Whereas at lower temperatures, there are no significant differences in the shape of the EINS curves between the polymorphs (Figure 5A), at higher temperatures above 240 K (Figures 5B,C), the scattering intensity is higher in the low-Q region and lower in the high-Q region for LP60 than for LP27, indicating that there are dynamical differences between the polymorphs. Therefore, as a next step, the MSPF analysis was carried out to extract dynamical information in more detail (see Eq. 2). Figures 6A,B show examples of the MSPF fits to the measured EINS curves. It is seen that the EINS curves are fitted well over all the Q values measured. The extracted MSPF and the measure of motional heterogeneity β are shown in Figures 6C,D, respectively. While the behavior of the MSPF turns out to be similar between the polymorphs as observed in the MSD, the β values of LP27 and LP60 show distinct behavior above 250 K. Based on the β values obtained, the distribution of the squared atomic position fluctuation ( $r^{2}$ ) is calculated at 306 K using Eq. 3 and the result is shown in Figure 6E. It is found that LP27 contains a much higher fraction of atoms moving with a squared amplitude less than 0.25 Å^2^ than LP60 and a lower fraction of atomic motions with higher amplitudes. These results suggest that the dynamical differences between the two polymorphs are not in the averaged amplitude of the atomic motions as observed for the MSD and the MSPF, but in the distribution of the amplitude of the atomic motions at temperatures higher than 250 K.

**FIGURE 7:**
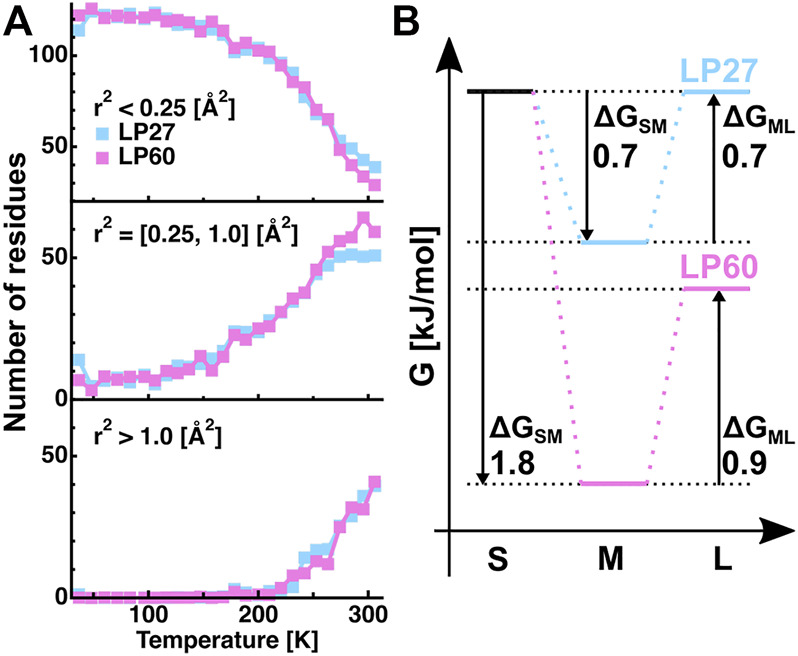
Analysis of the distributions of the squared position fluctuation obtained by the MSPF analysis. **(A)**: Temperature dependence of the number of residues undergoing motions with small [*r* ^2^ < 0.25 (Å^2^)], medium [0.25 (Å^2^) ≤ *r* ^2^ ≤ 1.0 (Å^2^)], and large amplitudes [1.0 (Å^2^) < *r* ^2^], which were calculated based on the β values of Figure 6D. **(B)**: Free energy differences between the states of motions of the small, medium, and large amplitudes, which were estimated from the number of residues contained in each state shown in Figure 7A at 306 K. “S”, “M”, and “L” denote the small, medium, and large amplitudes, respectively. ΔG_SM_ and ΔG_ML_ denote the free energy differences between the S and M states, and between the M and L states, respectively. The numerical values of ΔG_SM_ and ΔG_ML_ are in the unit of kJ/mol. The free energy of the fraction of atoms with small amplitudes of LP27 is set to be the same as that of LP60.

In order to further analyze the features of the distribution of the amplitudes, the $r^{2}$ values in Figure 6E were divided into three groups: a fraction of atomic motions with small amplitudes $r^{2}$ [ $r^{2}$ < 0.25 (Å^2^)], one of medium amplitudes [0.25 (Å^2^) ≤ $\;r^{2}$ ≤ 1.0 (Å^2^)], and one of large amplitudes [1.0 < $\;r^{2}$ (Å^2^)]. In the following, these three categories are referred to as “S”, “M”, and “L”, respectively. Then, the distribution of the amplitudes was integrated over $r^{2}$ contained in each of the three categories and the integrated values were calculated as a function of temperature, followed by normalization by the total number of residues in HEWL (129 residues). The results are shown in Figure 7A. It is found that at lower temperatures, almost all residues (∼120 residues) undergo motions with small amplitudes in both LP27 and LP60 while at higher temperatures above 250 K, LP60 shows less amounts of residues (∼10 residues) undergoing motions with small amplitudes. Regarding the motions with the medium amplitudes, these fractions show a behavior almost opposite to those with the small amplitudes: at higher temperatures, ∼10 more residues are involved in this motion in LP60 than in LP27. As for the motions with the large amplitudes, they emerge above 200 K, but there is no difference in behavior between the two polymorphs. Thus, the number of residues (hydrogen atoms) that occupies the S and M states is different between LP27 and LP60 above 250 K. Based on these fractions, the free energy difference (ΔG) at 306 K between the S and M states (ΔG_SM_), and between the M and L states (ΔG_ML_) is estimated from the relation N_2_/N_1_ = exp (−ΔG/RT), where N_1_ and N_2_ denote the number of atoms included in each category, R is the gas constant, and T is the absolute temperature. The resultant ΔG values are shown in Figure 7B. It is found that the M state is stabilized the most in both polymorphs, and that ΔG_SM_ of LP60 is about two times lower than that of LP27. These results imply that LP60 contains some atoms whose motion is more stabilized than that of LP27.

### QENS Measurements

From the EINS measurements described in the previous section, the dynamical differences between LP27 and LP60 were found to exist in the diffusive motions. Therefore, as a next step, in order to further investigate the nature of the differences in the diffusive motions between the polymorphs, QENS experiments were carried out. First, to see the possible difference in the QENS spectra between the polymorphs, S(ω) , which is obtained by the integration of  S(Q, ω) along Q, was calculated and compared as shown in Figure 2A. As evident from the ratio of S(ω) of the samples, the QENS spectrum of LP60 is broader than LP27. Moreover, both spectra show a larger width than the resolution function. These observations already suggest a difference in the diffusive nature between the polymorphs. The detailed analysis of the QENS spectra was then conducted by a global fitting approach based on the phenomenological equation (Eq. 5).

**FIGURE 8:**
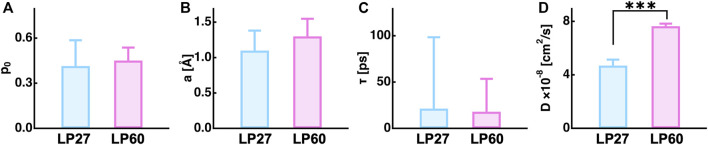
Summary of the dynamics parameters obtained by a global fitting to the measured QENS spectra. **(A)**, **(B)**, **(C)**, and **(D)** denote the fraction of the immobile atoms, the amplitude, the residence time, and the jump diffusion coefficient of atomic motions. The asterisks (***) show that the difference between the two values are statistically significant by Student’s *t*-test (*p* < 0.001).

Dynamics parameters extracted from the global fitting are summarized in Figure 8 (also tabulated in Table 1). It is found that the immobile fraction ( $\;p_{0}$ ) and the radius of the sphere ( a ) are the same within the errors between the polymorphs, suggesting that the averaged geometry of atomic motions is similar for both polymorphs. This is consistent with the EINS results that the MSD and MSPF values are similar between LP27 and LP60. Regarding the diffusive nature, whereas there is no difference in residence time between the polymorphs, the jump diffusion coefficient was found to be much larger for LP60 than for LP27 (*p* < 0.001 by Student’s *t*-test), indicating that the atoms of LP60 undergo enhanced diffusive motions compared with those of LP27. The broadening of the width of the Q-summed QENS spectra  S(ω) of LP60 compared with that of LP27 shown in Figure 2A is thus attributed to the increase in the jump diffusion coefficient. The present EINS and QENS measurements suggest that LP60 possesses a larger fraction of atoms undergoing diffusive motions with larger amplitudes than LP27 and that the atoms of LP60 are moving more rapidly than those of LP27.

**TABLE 1:** Summary of the dynamics parameters obtained by the global fitting to the QENS spectra. The values in the parentheses are errors associated with the fitting.

	*p*_0_	a [Å]	τ [ps]	D × 10^−8^ [cm^2^/s]
LP27	0.41 (0.17)	1.1 (0.3)	21.4 (77)	4.7 (0.4)
LP60	0.45 (0.09)	1.3 (0.2)	18.0 (35)	7.6 (0.2)

## Discussion

### Impact of Dynamics of Polymorphs on Their Physicochemical Properties

In this study, sub-ns dynamics of the lysozyme polymorphic fibrils termed LP27 and LP60 was investigated using EINS and QENS. The analysis shows that motional heterogeneity (the degree of the distribution of amplitudes) is lower for LP60 than for LP27 so that LP60 possesses a larger fraction of atoms undergoing diffusive motions with a larger amplitude than LP27. Moreover, the local diffusion of the atoms of LP60 was found to be enhanced compared to LP27. Thus, the present data show that the molecular dynamics of LP60 is enhanced compared with that of LP27.

The MSD and MSPF values at 306 K were 0.6–0.7 Å^2^ and ∼0.86 Å^2^, respectively, for both polymorphs. According to the previous studies on EINS using the same spectrometer as in this study, well-folded proteins such as alpha-lactalbumin and human acetylcholinesterase have the MSD and MSPF values of 0.7–0.8 Å^2^ and 0.8–1.6 Å^2^, respectively, at around 300 K at the same hydration ratio as in this study (Peters et al., 2016; Zeller et al., 2018). This suggests that the polymorphs studied here fall into a category of relatively rigid molecules. Looking at the distribution of amplitudes between the polymorphs (Figure 7A), the main difference lies in the small and medium amplitudes (the S and M states, respectively) without changing the number of residues classified as the large amplitudes (the L state). These results indicate that only small-scale local motions are involved in the dynamical differences between the two polymorphs. This is consistent with the fact that the differences in the EINS spectra are observed mainly in the higher-Q region (Figure 5).

The fraction of immobile atoms is attributed to amino acids in the core of the protein and the fraction of mobile atoms is attributed to residues located closer to or on the protein surface (Stadler et al., 2014, 2016) because the motions of atoms and thus side-chains in the protein core are much more constrained and slower than those on the protein surface (Dellerue et al., 2001). The fraction of immobile atoms was ∼0.43 for both samples (Figure 8), which corresponds to ∼296 (= 689 × 0.43) nonexchangeable hydrogen atoms (note that 689 is the total number of nonexchangeable hydrogen atoms of HEWL). Assuming that the average number of nonexchangeable hydrogen atoms in an amino acid is 6, this corresponds to 49 residues, which is in line with 51 residues, the number of residues forming the core region of lysozyme amyloid fibrils formed at pH 2.0 (Frare et al., 2004). Although it is likely that the core region is not identical among polymorphs, it is reasonable to assume that the immobile atoms are mainly located in the core region of the polymorphs. Then, the dynamical parameters of mobile atoms, i.e., the residence time and jump diffusion coefficient, mainly derive from the motions of non-core regions, which are closer to the surface of the polymorphs and tend to be exposed to the solvent. The larger jump diffusion coefficient for LP60 indicates that the side chains are less constrained during the jump motion and can move more easily than LP27. It is thus likely that the non-core regions are more flexible for LP60 than for LP27. In addition, since increase in the conformational freedom of protein molecules generally enhances binding kinetics (Shoemaker et al., 2000), LP60 would be able to have more opportunities to interact with other molecules than LP27.

Enhanced dynamics of LP60 compared with LP27 offers an explanation on morphological differences observed for lysozyme polymorphs formed at near-neutral pH or acidic pH. Since LP27 contains atoms less mobile than LP60, the interactions between monomers would be strong enough to maintain long fibrils. On the other hand, in the case of LP60, more mobile atoms than LP27 would weaken the inter-monomer interactions within the fibrils, which would destabilize the fibrils, leading to shorter fragments of fibrils. Moreover, it is likely that the atoms moving more rapidly with larger amplitudes in LP60 facilitate conformational adjustments for the interactions between fibrils, which would promote non-specific binding and the aggregation of the short fibrils as shown in Figure 3F.

In a recent cross-seeding measurement, it has been shown that both of the following two types of solution conditions, where 1) fibrils are formed at pH 6.3 from human lysozyme monomers and HEWL seeds preformed at pH 2.0 or 2) fibrils are formed at pH 2.0 from both human lysozyme monomers and HEWL seeds preformed at pH 6.3, lead to the fibrils with the same morphology as those formed at pH 6.3 (Rahimi Araghi and Dee, 2020). These results indicate that polymorphs formed at near-neutral pH have the ability to promote more monomer binding than those formed at acidic pH. To explain these observations, it has been proposed that the configurational dynamics of polymorphs formed at near-neutral pH might be enhanced compared with those formed at acidic pH (Rahimi Araghi and Dee, 2020). Our current results support this notion and also imply the importance of sub-ns protein dynamics as a determinant of the physicochemical properties of the proteins.

### Dynamical Differences Between Lysozyme Polymorphs

The current EINS and QENS results show that the sub-ns dynamics is enhanced for LP60 compared with LP27. A likely origin of this observation is that the residues which comprise the core of the lysozyme fibrils are different between LP27 and LP60. A core-forming region of lysozyme amyloid fibrils formed at pH 2.0 has been determined to be the residues 57–107 (Frare et al., 2004). Although the condition of the fibril formation is different from the one employed here, it is probable that the core-forming region shifts according to the fibrilization conditions. Since some charged or polar residues are distributed in the proximity of the residues 57–107, the shifts in the core-forming region would alter the physicochemical properties of other regions closer to the polymorph surface and exposed to the solvent, leading to the modulation of fluctuations of the amino acid side chains. Although the structural analysis of lysozyme polymorphs has not yet been carried out, different molecular structures between polymorphic fibrils have indeed been observed in another amyloidogenic protein, amyloid-β (Petkova et al., 2005). In addition to the shift in the core-forming region described above, it is also likely that the physical properties of hydration water are not the same between LP27 and LP60. It has been shown that, in general, the density and the mobility of the hydration water vary depending on the charge distribution on the protein surface (Matsuo et al., 2013a, 2016; Kim et al., 2016). Thus, if the non-core regions contain different charged and/or polar residues depending on the polymorphs, the hydration water mobility around these regions could be modulated. Furthermore, the mobility of hydration water around tau protein has been shown to be enhanced upon amyloid fibril formation (Fichou et al., 2015), suggesting that the difference in the residues exposed to the solvent leads to the difference in hydration water mobility. Since the hydration water molecules, in particular those in the first hydration shell, directly interact with the amino acid side chains on the protein surface, polymorph-specific interplay between the hydration water and side chains would determine the motions of both hydration water and side chains unique to each polymorph.

In this study, hydrated powder samples were employed in order to focus on only local atomic motions. Thus, any larger-scale motions, e.g., diffusion of the segments in the fibrils and the bending motions of the entire fibrils, are suppressed and no dynamical information is obtained here regarding these motions. It is, however, most likely that the differences in the local atomic motions between the polymorphs observed in this study lead to modulation of the large-scale motions. Therefore, in the future, it is essential to investigate the molecular dynamics of the polymorphs in aqueous solutions in a wide range of time- and space-scales, and then integrate all the findings to profoundly understand the amyloid polymorphism and eventually pathogenesis of lysozyme amyloidosis. This could partly be achieved by EINS/QENS measurements using spectrometers with different energy resolutions (different time windows) and the neutron spin-echo spectroscopy (Gardner et al., 2020), which provides dynamical information occurring at much longer time scale (several hundred nanoseconds) than typical QENS instruments.

This study has succeeded to detect small but significant differences in sub-nanosecond protein dynamics between polymorphs from the same protein. Dynamical modulation caused by alteration of the protein properties has been studied also for other systems using neutron scattering: a previous study using EINS and QENS has shown a dynamical change of the bacterial reaction center (RC) proteins caused by non-functional two point mutations (Sacquin-Mora et al., 2007). Recently, even a single point mutation has been found to modulate the dynamics of proteins such as human myelin protein P2 (Laulumaa et al., 2015) and human cardiac troponin (Matsuo et al., 2013b, 2017). These results, combined with the current study, demonstrate the sensitivity of incoherent neutron scattering to detect even slight changes in molecular dynamics induced by external or internal environmental perturbations.

It is also worth noting that polymorphs formed at near-neutral pH are more cytotoxic than those formed at acidic pH (Mossuto et al., 2010; Mocanu et al., 2014). Although there are several types of mechanisms of cytotoxicity (Butterfield, 2002), it has been shown that shorter fibrils of β_2_-microgloburin interact more strongly than longer fibrils with lipid membranes and then cause stronger membrane disruption (Milanesi et al., 2012), enhancing cytotoxicity (Xue et al., 2009). It is thus conceivable that different molecular motions of polymorphs modulate the rate of binding to and interactions with membranes. Therefore, knowledge of the molecular dynamics of polymorphs would serve as a basis to ultimately elucidate the molecular mechanism of cytotoxicity caused by fibril-membrane interactions.

To the authors’ knowledge, this is the first study to characterize the differences in molecular dynamics of polymorphic protein aggregates. The present results imply that lysozyme amyloid fibrils show not only polymorphism, but also dynamical diversity (it may be coined as “polydynamism”). Since amyloid polymorphism is generally considered to be closely related to the severity of symptoms of amyloidosis, and since physicochemical properties of polymorphs are sensitive to the chemical environment in which they are formed, integration of the structural and dynamical knowledge obtained by future systematic studies on various kinds of polymorphs would definitely advance our understanding of the molecular mechanism of various diseases not limited to lysozyme amyloidosis.

## Data Availability

The original contributions presented in the study are included in the article/Supplementary Material, further inquiries can be directed to the corresponding authors.

## Abbreviations

LP27, Lysozyme polymorph formed at pH 2.7; LP60, Lysozyme polymorph formed at pH 6.0; EINS, Elastic incoherent neutron scattering; QENS, Quasielastic neutron scattering; iNS, Incoherent neutron scattering.
